# Workaholism among nurses in China: a nationwide cross-sectional survey

**DOI:** 10.1186/s12912-025-03455-5

**Published:** 2025-07-01

**Authors:** Yan Gao, Hongmin Ye, Shuqin Hong, Xue Bai, Xiuni Gan

**Affiliations:** 1 Nursing Department, The Second Affiliated Hospital of Chongqing Medical University, Chongqing, China; 2 School of Nursing, Chongqing Medical University, Chongqing, China; 3 Health Management Centre, The Second Affiliated Hospital of Chongqing Medical University, Chongqing, China

**Keywords:** Mental health, Nurses, Occupational health, Work engagement

## Abstract

**Background:**

Anxiety and depression among nursing staff, stemming from difficulties in balancing high-pressure, high-load work with daily life, are on the rise. This phenomenon may be linked to workaholism among clinical nurses, yet the prevalence of workaholism among Chinese nurses remains underreported.

**Objectives:**

To investigate the current state of workaholism among Chinese nurses and identify factors influencing it.

**Methods:**

We distributed study information and a survey link via WeChat to professional nursing networks. The survey included demographic characteristics and the Dutch Work Addiction Scale (DUWAS). Descriptive and comparative analyses were performed, and multiple regression analysis was performed using the workaholism score as the dependent variable.

**Results:**

A total of 3,596 Chinese registered nurses participated in the survey. The mean workaholism total score was 29.87 (*SD* = 4.93), with an average item score of 2.99 (*SD* = 0.49). Regression analysis revealed that education level (*β*=0.05), position (*β*=0.11), professional title (*β*=0.10), weekly night shifts (*β*=0.07), and weekly work hours (*β*=0.15) were positively associated with workaholism. Additionally, compared to nurses without children, those with children (*β*=0.06) exhibited higher levels of workaholism (all *P* < 0.05).

**Conclusions:**

Chinese nurses exhibit moderate levels of workaholism. Nursing managers can enhance occupational well-being and reduce workaholism by implementing targeted interventions focused on key factors such as educational background, professional title, position, weekly night shift frequency, average weekly working hours, and parental status.

**Clinical trial number:**

Not applicable.

**Supplementary Information:**

The online version contains supplementary material available at 10.1186/s12912-025-03455-5.

## Introduction

The rapid expansion of the healthcare industry in response to increasing health service demands has posed significant work challenges for healthcare workers. The psychological state of nursing staff is a major concern, with surveys indicating that 54.8% of nurses in China exceed 8 hours of daily work [1]. Additionally, phenomena such as ‘karoshi’ ‘depression’ and ‘high cancer incidence’ have become increasingly prominent among Chinese nurses. Some studies have shown that anxiety and depression resulting from nurses’ difficulty in balancing high-pressure, high-load work with daily life show a rising trajectory, and this phenomenon may be related to clinical nurses’ workaholism [2].

## Background

Workaholism was first described in 1971 by Oates as “an addiction to work, characterized by an uncontrollable compulsion to work excessively” [3]. Since this concept was put forward, a succession of scholars have studied workaholism from different perspectives. Initially, Mosier [4] defined work addicts solely by their work hours, i.e., people who work for over 50 hours per week without any breaks for recreation. In the 1990s, Spence and colleagues [5] proposed the Three-Dimensional Theory of Work Addiction, which categorizes work addiction into three dimensions: high work engagement, high compulsive drive, and low work enjoyment. Schaufeli et al. [6] added an attitude and asserted that work addicts tend to work excessively and become compulsive preoccupation with work, neglecting their personal life. In today’s competitive society, where employees invest significant time and energy into their work [7], the risk of workaholism is rising. Numerous studies have highlighted the negative consequences of workaholism, including impaired physical and mental health [8], decreased work and life satisfaction [9], work-family conflict [10], burnout [11], and performance deterioration [12].

Workaholism is a complex phenomenon that may be triggered by individual, organizational, and sociocultural factors [13, 14]. Some countries have already focused on the phenomenon of workaholism among nurses. In a survey of 278 Korean nurses [15], it was found that 30.9% of nurses had a moderate level of workaholism and 15.5% high workaholism. About 28.5% of Japanese nurses have workaholic tendencies [16]. Additionally, a comprehensive study involving ten countries on factors related to workaholism in nurses’ mental health pointed out that the factors associated with workaholism include burnout, stress, anxiety, depression, and sleep-related issues [17]. Working day and night and constantly thinking about work problems can compromise the physical health of work addicts [16], leading to declining mental health [18], reduced quality of occupational life [19], and strained relationships with family and friends. Collectively, these factors ultimately result in lower social relationship quality and work-family conflicts. Furthermore, empirical evidence has demonstrated significant associations between workaholism and work-related adverse events (e.g., patient/other harm or near-harm events) [20], suggesting that workaholic tendencies may jeopardize patient safety outcomes.

Most studies on workaholism in China have focused on corporate employees and college teachers, with limited research into its effects among nurses [21, 22]. Given the high prevalence of workaholism among nurses and its impact on burnout and patient care, there is an urgent need to identify effective interventions. This study aimed to investigate the current status of workaholism among nurses in China and analyze its influencing factors, to develop an evidence-based framework to improve nurses’ well-being, stabilize the nursing workforce, and reduce patient safety risks in clinical practice.

## Methods

In this observational cross-sectional study, we distributed the questionnaire via an online survey platform in China (https://www.yibiaoda.com/*).* This study adhered to the Equator Network’s Checklist for Reporting Results of Internet E-Surveys (CHERRIES) [23].

### Ethical statement

The study protocol was reviewed and approved by the Second Affiliated Hospital of Chongqing Medical University (Aug. 1, 2024; Approval No. 2024-52). Participants were informed on the first page of the survey that submitting the survey constitutes their consent to participate in the study. Participants were provided with detailed information about the study, including its purpose, content, estimated completion time (3–5 min), secure data storage procedures, and contact information for the researchers. Due to the anonymity of the responses, participants were unable to withdraw from the study after submission.

### Sample and sampling

Registered nurses were recruited via convenience sampling from 16 provinces and 59 cities across North, East, South, Central, Northeast, Southwest, and Northwest China, ensuring geographical representation. The sample included diverse healthcare institutions and departments, such as general hospitals, specialty hospitals, community hospitals, and mental health centers. Inclusion criteria: (1) age between 18 and 60 years; (2) registered nurses; (3) willingness to participate with informed consent. Exclusion criteria: (1) intern nurses; (2) nurses in advanced training. The study employed multiple linear regression analysis, which requires a sample size of 5 to 10 times the number of predictors [24]. The study included 24 predictors. Considering a 20% attrition rate, the required sample size was at least 144 cases. The final sample included 3596 cases.

### Data collection

From September 22, 2023 to March 20, 2024, researchers distributed the survey link nationwide through nursing academic societies. Members forwarded the link to hospital nursing networks and continuing education groups within their jurisdictions for participation. The researchers initially used WeChat to distribute a QR code linking to an online survey page, allowing participants to complete the survey using their mobile phones, tablets, or computers. Participants were limited to one response per device (computer or mobile phone). WeChat is a mobile-based social networking application similar to WhatsApp. As of the end of 2018, WeChat had over 1 billion daily active users [25]. WeChat-based questionnaires have been increasingly adopted in various fields, including social sciences, medicine, and nursing, and have been well received by respondents [26].

### Survey, variables, and instruments

Through preliminary literature review and research team discussions, the self-designed general information questionnaire collected data on the following variables: gender, age, highest level of education, hospital level, position, employment type, professional title, years of work experience, whether they have children, marital status, political affiliation, monthly salary, weekly night shifts, and weekly work hours.

The Dutch Work Addiction Scale (DUWAS), developed by Schaufeli and revised by Linlin Zhang et al. [27], was employed. The scale comprises 10 items divided into two subscales: Working Excessively (WE) and Working Compulsively (WC), each containing 5 items. A four-point Likert scale was used to assess DUWAS, with higher scores indicating a greater degree of workaholism. The original scale demonstrated Cronbach’s α coefficients of 0.73–0.78 for WE and 0.68–0.78 for WC [28]. The DUWAS has been applied and validated in several countries, including Brazil [29], Spain [30], Argentina [31], Italy [32], and Norway [33], demonstrating good psychometric properties and ease of use.

### Statistical analyses

IBM SPSS Statistics version 27 (IBM Corp.) was used for the statistical analyses. Categorical data were expressed as frequencies and percentages, while continuous data were expressed as mean and standard deviation(SD). For univariate analysis, we used t-tests and ANOVA. In multivariable linear regression analysis, only variables that were significant in univariate analysis were included as independent variables, with nurses’ workaholism scores as the dependent variable.

Owing to the mandatory nature of all questionnaires, there were no missing data in this study. Probability plots and values of skewness and kurtosis were used to assess the normality of the dependent variable. The probability plots are shown in Supplementary file 1. The normality thresholds were defined as |skewness (S)| < 2 and |kurtosis (K)| < 4 [34]. The workaholism score (S =−0.347, K = 1.576) meet these criteria, indicating approximate normal distribution.

In linear regression, if independent variable was a continuous or ordered, it was directly included it in the model because its numercal magnitude quantifies the association with the dependent variable; if an independent variable was a categorical variable, dummy coding was applied because categorical values denote group membership rather than quantitative trends [35]. Variables requiring dummy coding included employment type (reference group: contract basis), parental status (reference: no children), marital status (reference: unmarried/divorced/widowed), and political affiliation (reference: other).

Multicollinearity was assessed using the correlation coefficient between independent variables (> 0.7) as the exclusion threshold, variance inflation factor (VIF) (> 10), and tolerance (< 0.2) [36]. When multicollinearity was detected, separate analyses were conducted, and variables with the highest adjusted R² values (indicating the best model fit in explaining variance in the dependent variable) were retained. Standardized beta coefficients (*β*) were used to assess the relative importance of each predictor, as the included variables were measured in different units [37]. A P-value less than 0.05 was considered indicative of statistical significance.

## Results

### Response to the questionnaire survey

A total of 4,431 nurses participated in the survey, with 3,600 completing the questionnaire (completion rate: 81.2%). One response was completed by a researcher for software testing; two respondents were older than 60 years; and one was younger than 18 years. Consequently, 3,596 responses met the eligibility criteria and were included in the analysis. The average completion time was 6 min and 16 s.

### Demographic characteristics

The demographic characteristics of the participants are summarized in Table 1. Most nurses were female (*n* = 3,419, 95.1%), with a mean age of 32.58 years (*SD* = 7.31). Of these, 85.6% (*n* = 3,078) were aged between 21 and 40 years. Nearly halfs (47.8%, *n* = 1,718) were from 3A hospitals. Additionally, 69.7% (*n* = 2,507) were married, 63.3% (*n* = 2,278) had children, and 85.8% (*n* = 3,086) were charge nurses. Furthermore, 83.0% (*n* = 2,986) held a bachelor’s degree, while 2.3% (*n* = 84) had a master’s degree or higher(including doctoral degrees).

**Table 1:** Demographic characteristics of the participating nurses (*n* = 3596)

Demographic	Options	No. of participants, *n*	%
**Gender**	Male	177	4.9
	Female	3419	95.1
**Age (years)**	18～20	10	0.3
	21～30	1627	45.2
	31～40	1451	40.4
	41～50	404	11.2
	51～60	104	2.9
**Education**	Diploma/Associate’s Degree	526	14.6
	Bachelor’s degree	2986	83.0
	Master’s degree or above	84	2.3
**Hospital level**	3 A hospital	1718	47.8
	3B hospital	567	15.8
	2 A hospital	1055	29.3
	2B hospital	85	2.4
	Others	171	4.8
**Position**	Nurse	3086	85.8
	Assistant nurse manager	45	1.3
	Head nurse	362	10.1
	Section nurse manager	50	1.4
	Deputy director of nursing	9	0.3
	Director of nursing	44	1.2
**Type of employment**	Staffing of public institution	851	23.7
	Contract basis	2745	76.3
**Title**	Junior (Nurse or Senior Nurse)	2050	57.0
	Intermediate (Nurse in Charge)	1290	35.9
	Senior (Nurse Director or Nurse Deputy Director)	256	7.1
**Working experience (years)**	<1	166	4.6
	1～5	887	24.7
	6～10	965	26.8
	11～19	1110	30.9
	≥ 20	468	13.0
**Having children**	No	1318	36.7
	Yes	2278	63.3
**Marital status**	Married	2507	69.7
	Single/Divorced/Widowed	1089	30.3
**Political affiliation**	Members of the Communist Party of China	520	14.5
	Members of the Communist Youth League of China	853	23.7
	Mass	2134	59.3
	Others	89	2.5
**Monthly wage (CNY)**	≤ 4 000	596	16.6
	4 001～8 000	1835	51.0
	8 001～12 000	850	23.6
	≥ 12 001	315	8.8
**Night shifts per week**	0	1177	32.7
	1	757	21.1
	2	970	27.0
	≥ 3	692	19.2
**Weekly work hours (h)**	≤ 40	740	20.6
	41～48	1998	55.6
	>48	858	23.9

### Nurses’ workaholism score and univariate analysis

The total score for workaholism across all dimensions was 29.87 (*SD* = 4.93), with an average score of 2.99 (*SD* = 0.49), indicating that moderate workaholism level among Chinese nurses. Scores for the WE dimension ranged from 4 to 20, with a total score of 15.31 (*SD* = 3.02) and a mean score of 3.06 (*SD* = 0.60). Scores for the WC dimension ranged from 4 to 20, with a total score of 14.57 (*SD* = 2.63) and a mean score of 2.91 (*SD* = 0.53).

As summarized in Table 2, univariable analysis identified significant associations (*P* < 0.05) between workaholism and the following variables: age, education, hospital level, position, employment type, professional title, years of work experience, whether they have children, marital status, political affiliation, monthly salary, weekly night shifts, and weekly work hours.

**Table 2:** Nurses’ workaholism score and univariable analysis (*n* = 3596)

	*n*	Total score($\bar{\text{x}}$ ± S)	Mean scors($\bar{\text{x}}$ ± S)	WE($\bar{\text{x}}$ ± S)	WC ($\bar{\text{x}}$ ± S)
**Gender**					
Male	177	30.11± 5.03	3.01±0.50	3.13 ± 0.60	2.90 ± 0.53
Female	3419	29.86± 4.92	2.99±0.49	3.06 ± 0.60	2.91 ± 0.53
*t/F*		0.671	0.671	1.480	-0.443
*P* value		0.502	0.502	0.139	0.658
**Age (years)**					
18～20	10	30.30± 3.47	3.03±0.35	3.14 ± 0.46	2.92 ± 0.29
21～30	1627	29.19± 4.75	2.92±0.47	3.01 ± 0.60	2.82 ± 0.51
31～40	1451	30.43± 4.86	3.04±0.49	3.10 ± 0.59	2.98 ± 0.52
41～50	404	30.52± 5.01	3.05±0.50	3.10 ± 0.61	2.98 ± 0.52
51～60	104	30.15± 6.62	3.02±0.66	3.02 ± 0.73	3.01 ± 0.66
*t/F*		14.551	14.551	5.013	21.988
*P* value		<0.001^***^	<0.001^***^	<0.001^***^	<0.001^***^
**Education**					
Diploma/Associate’s Degree	526	28.60± 5.24	2.86±0.52	2.91 ± 0.64	3.28 ± 0.55
Bachelor’s degree	2986	30.05± 4.84	3.01±0.48	3.08 ± 0.59	2.93 ± 0.52
Master’s degree or above	84	31.37± 4.51	3.14±0.45	3.28 ± 0.55	3.00 ± 0.54
*t/F*		23.855	23.855	24.747	11.933
*P* value		<0.001^***^	<0.001^***^	<0.001^***^	<0.001^***^
**Hospital level**					
3 A hospital	1718	30.05± 4.90	3.00±0.49	3.08 ± 0.60	2.93 ± 0.53
3B hospital	567	29.73± 5.10	2.97±0.51	3.06 ± 0.61	2.89 ± 0.55
2 A hospital	1055	29.87± 4.78	2.99±0.48	3.06 ± 0.61	2.92 ± 0.50
2B hospital	85	27.74± 5.57	2.77±0.56	2.84 ± 0.68	2.71 ± 0.56
Others	171	29.66± 4.85	2.97±0.48	3.05 ± 0.60	2.88 ± 0.51
*t/F*		4.725	4.725	3.386	4.166
*P* value		<0.001^***^	0.001^**^	0.009^**^	0.002^**^
**Position**					
Nurse	3086	29.54± 4.86	2.95±0.49	3.03 ± 0.61	2.88 ± 0.52
Assistant nurse manager	45	31.18± 5.08	3.12±0.51	3.24 ± 0.58	3.00 ± 0.60
Head nurse	362	31.83± 4.68	3.18±0.47	3.26 ± 0.53	3.10 ± 0.51
Section nurse manager	50	31.02± 5.64	3.10±0.56	3.18 ± 0.65	3.03 ± 0.61
Deputy director of nursing	9	33.67± 5.43	3.37±0.54	3.47 ± 0.53	3.27 ± 0.63
Director of nursing	44	33.41± 4.41	3.34±0.44	3.40 ± 0.54	3.27 ± 0.45
*t/F*		21.565	21.565	15.025	17.898
*P* value		<0.001^***^	<0.001^***^	<0.001^***^	<0.001^***^
**Type of employment**					
Staffing of public institution	851	30.55± 5.00	3.06±0.50	3.13 ± 0.61	2.98 ± 0.53
Contract basis	2745	29.66± 4.88	2.97±0.49	3.04 ± 0.60	2.89 ± 0.52
*t/F*		4.635	4.635	3.975	4.102
*P* value		<0.001^***^	<0.001^***^	<0.001^***^	<0.001^***^
**Title**					
Junior (Nurse or Senior Nurse)	2050	29.22± 4.82	2.92±0.48	3.00 ± 0.61	3.25 ± 0.61
Intermediate (Nurse in Charge)	1290	30.51± 4.80	3.05±0.48	3.11 ± 0.58	2.99 ± 0.52
Senior (Nurse Director or Nurse Deputy Director)	256	31.89± 5.38	3.19±0.54	3.25 ± 0.61	3.13 ± 0.55
*t/F*		51.913	51.913	26.587	56.896
*P* value		<0.001^***^	<0.001^***^	<0.001^***^	<0.001^***^
**Working experience (years)**					
<1	166	28.72± 4.18	2.87±0.42	2.96 ± 0.53	2.79 ± 0.46
1～5	887	29.00± 4.80	2.90±0.48	3.00 ± 0.62	2.80 ± 0.52
6～10	965	29.83± 4.72	2.98±0.47	3.08 ± 0.59	2.89 ± 0.51
11～19	1110	30.42± 4.97	3.04±0.50	3.09 ± 0.60	2.99 ± 0.53
≥ 20	468	30.72± 5.36	3.07±0.54	3.12 ± 0.63	3.03 ± 0.55
*t/F*		16.437	16.437	5.365	25.568
*P* value		<0.001^***^	<0.001^***^	<0.001^***^	<0.001^***^
**Having children**					
No	1318	29.15± 4.74	2.91±0.47	3.02 ± 0.61	2.81 ± 0.51
Yes	2278	30.29± 4.98	3.03±0.50	3.08 ± 0.60	2.97 ± 0.52
*t/F*		-6.827	-6.827	-2.944	-9.263
*P* value		<0.001^***^	<0.001^***^	<0.001^***^	<0.001^***^
**Marital status**					
Married	2507	30.16± 4.94	3.02±0.49	3.08 ± 0.60	2.96 ± 0.52
Single/Divorced/Widowed	1089	29.21± 4.83	2.92±0.48	3.03 ± 0.61	2.82 ± 0.52
*t/F*		5.362	5.362	2.328	7.373
*P* value		<0.001^***^	<0.001^***^	0.020^*^	<0.001^***^
**Political affiliation**					
Members of the Communist Party of China	520	30.88± 4.97	3.09±0.48	3.17 ± 0.57	3.01 ± 0.52
Members of the Communist Youth League of China	853	29.17± 4.62	2.92±0.46	3.02 ± 0.59	2.81 ± 0.50
Mass	2134	29.84± 5.02	2.98±0.50	3.02 ± 0.59	2.92 ± 0.53
Others	89	31.49± 4.95	3.15±0.50	3.23 ± 0.63	3.07 ± 0.53
*t/F*		16.562	16.562	9.637	19.734
*P* value		<0.001^***^	<0.001^***^	<0.001^***^	<0.001^***^
**Monthly wage (CNY)**					
≤ 4 000	596	29.02± 5.02	2.90±0.50	2.97 ± 0.63	2.83 ± 0.53
4 001～8 000	1835	29.76± 4.88	2.98±0.49	3.05 ± 0.60	2.90 ± 0.52
8 001～12 000	850	30.24± 4.90	3.02±0.49	3.11 ± 0.60	2.93 ± 0.53
≥ 12 001	315	31.41± 4.73	3.11±0.47	3.18 ± 0.55	3.05 ± 0.52
*t/F*		15.055	15.055	10.701	12.865
*P* value		<0.001^***^	<0.001^***^	<0.001^***^	<0.001^***^
**Night shifts per week**					
0	1177	30.06± 5.10	3.01±0.51	3.05 ± 0.61	2.96 ± 0.53
1	757	29.49± 4.80	2.95±0.48	3.03 ± 0.59	2.87 ± 0.53
2	970	29.64± 4.72	2.96±0.47	3.06 ± 0.60	2.87 ± 0.51
≥ 3	692	30.29± 5.00	3.03±0.50	3.12 ± 0.61	2.94 ± 0.53
*t/F*		4.484	4.484	3.031	8.318
*P* value		0.004^**^	0.004^**^	0.028^*^	<0.001^***^
**Weekly work hours (h)**					
≤ 40	740	28.44± 4.71	2.84±0.47	2.84 ± 0.58	2.85 ± 0.50
41～48	1998	29.90± 4.81	2.99±0.48	3.07 ± 0.58	2.91 ± 0.52
>48	858	31.04± 5.04	3.10±0.50	3.23 ± 0.62	2.98 ± 0.55
*t/F*		57.101	57.101	86.999	12.184
*P* value		<0.001^***^	<0.001^***^	<0.001^***^	<0.001^***^

WE, Working Excessively; WC, Working Compulsively

*F*, ANOVA; *t*, t-test

⁎⁎⁎ *P* ≤ 0.001

⁎⁎ *P* ≤ 0.01

⁎ *P* < 0.05

### Results from the multiple regression analysis

The mean score of the nurses’ workaholism was the dependent variable, while the factors found to be statistically significant in the univariable analysis (i.e., age, education, hospital level, position, employment type, professional title, years of work experience, whether they have children, marital status, political affiliation, monthly salary, weekly night shifts, and weekly work hours) were used as independent variables in multiple linear regression analyses. The independent variables were assigned as shown in Table 3.

**Table 3:** Overview of the variables, their response options, and how responses were coded numerically for analyses

Variables	Survey response options	Coding for regression analyses
Age (years)	18～20,21～30,31～40,41～50,51～60	Kept as ordinal variable(i.e., 18～20 = 1,21～30 = 2,31～40 = 3,41～50 = 4,51～60 = 5)
Education	Diploma/Associate’s degree, Bachelor’s degree, Master’s degree or above	Kept as ordinal variable (i.e., Diploma/Associate’s degree = 1, Bachelor’s degree = 2, Master’s degree or above = 3)
Hospital level	3 A hospital, 3B hospital, 2 A hospital, 2B hospital, Others	Kept as ordinal variable (i.e., 3 A hospital = 1, 3B hospital = 2, 2 A hospital = 3, 2B hospital = 4, Others = 5)
Position	Nurse, Assistant nurse manager, Head nurse, Section nurse manager, Deputy director of nursing, Director of nursing	Kept as ordinal variable (i.e., Nurse = 1, Assistant nurse manager = 2, Head nurse = 3, Section nurse manager = 4, Deputy director of nursing = 5, Director of nursing = 6)
Type of employment	Staffing of public institution, Contract basis	Dichotomized into a dummy variable (i.e.,Staffing of public institution = 1, Contract basis = 0)
Title	Junior (Nurse or Senior Nurse), Intermediate (Nurse in Charge), Senior (Nurse Director or Nurse Deputy Director)	Kept as ordinal variable (i.e., Junior = 1, Intermediate = 2, Senior = 3)
Working experience (years)	<1, 1～5, 6～10, 11～19, ≥ 20	Kept as ordinal variable (i.e., <1 = 1;1～5 = 2；6～10 = 3；11～19 = 4；≥20 = 5)
Having children	No, Yes	Dichotomized into a dummy variable (i.e.,No = 0,Yes = 1)
Marital status	Married, Single/Divorced/Widowed	Dichotomized into a dummy variable (i.e.,Single/Divorced/Widowed = 0,Married = 1)
Political affiliation	Members of the Communist Party of China, Members of the Communist Youth League of China, Mass, Others	Dichotomized into a dummy variable (i.e.,Members of the Communist Party of China = 1, Members of the Communist Youth League of China = 0, Mass = 0, Others = 0)
Monthly wage (CNY)	≤ 4 000, 4 001～8 000, 8 001～12 000, ≥ 12 001	Kept as ordinal variable (i.e.,≤4 000 = 1, 4 001～8 000 = 2, 8 001～12 000 = 3, ≥ 12 001 = 4)
Night shifts per week	0, 1, 2, ≥3	Kept as ordinal variable (i.e.,0 = 1, 1 = 2, 2 = 3, ≥ 3 = 4)
Weekly work hours (h)	≤ 40, 41～48, >48 = 3	Kept as ordinal variable (i.e.,≤40 = 1, 41～48 = 2, >48 = 3)

The results revealed several significant predictors of workaholism among nurses (Table 4). Specifically, weekly work hours (*β* = 0.15, *P* < 0.05), followed by position (*β* = 0.11, *P* < 0.05) and professional title (*β* = 0.10, *P* < 0.05). Night shifts per week (*β* = 0.07, *P* < 0.05), education level (*β* = 0.05, *P* < 0.05), and parenthood status (*β* = 0.06, *P* < 0.05; reference: childless nurses) also showed statistically significant positive effects on workaholism.

**Table 4:** Multiple regression analyses, using nurses’ workaholism mean score of as the dependent variables

	Unstandardized coefficients		Standardized coefficients	t	*P*	VIF
	B	Std. error	β			
Constant	2.35	0.07		32.99	<0.001^***^	
Age (years)	-0.04	0.02	-0.06	-1.92	0.055	3.91
Education	0.06	0.02	0.05	2.94	0.003^**^	1.18
Hospital level	-0.01	0.01	-0.030	-1.65	0.099	1.32
Position	0.06	0.01	0.11	5.12	<0.001^***^	1.72
Title	0.08	0.02	0.10	3.77	<0.001^***^	2.55
Working experience (years)	0.02	0.02	0.05	1.49	0.136	4.40
Monthly wage (CNY)	0.02	0.01	0.031	1.54	0.123	1.53
Night shifts per week	0.03	0.01	0.07	3.99	<0.001^***^	1.32
Weekly work hours (h)	0.11	0.01	0.15	9.32	<0.001^***^	1.04
Staffing of public institution	-0.01	0.02	-0.01	-0.48	0.633	1.55
Having children	0.06	0.03	0.06	2.27	0.023^*^	2.85
Married	-0.01	0.03	-0.01	-0.37	0.711	2.31
Members of the Communist Party of China	0.01	0.03	0.01	0.34	0.733	1.27
Members of the Communist Youth League of China	0.01	0.02	0.01	0.62	0.538	1.56
Others	0.09	0.05	0.03	1.76	0.078	1.04
R^2^				7.80		
adj. R^2^				7.40		
∆R^2^				7.80		
F				20.24		
*P*				<0.001^***^		

*β* = Standardized beta

⁎⁎⁎ *P* ≤ 0.001

⁎⁎ *P* ≤ 0.01

⁎ *P* < 0.05

## Discussion

Through a nationwide questionnaire survey across 16 provinces and 59 cities, we assessed workaholism among Chinese nurses using the 10-item Dutch Work Addiction Scale (DUWAS-10). The total workaholism score (sum of all items) was 29.87 (*SD* = 4.93), with a mean item score of 2.99 (*SD* = 0.49). These scores indicate a moderate level of workaholism, consistent with Asian nursing benchmarks [38]. Key predictors of higher workaholism scores included higher education (*β* = 0.05), senior positions (*β* = 0.11), advanced titles (*β* = 0.10), frequent occurrence of night shifts (*β* = 0.07), prolonged weekly work hours (*β* = 0.15), and parenthood status (*β* = 0.06).

The dimensional scores in the current study were consistent with those reported by Balducci et al. [39] and Mir et al. [40] among Portuguese nurses, with notably higher scores particularly observed in the excessive work dimension. This may be attributed to the current relative shortage and uneven distribution of nursing personnel in China [41], leading to heightened occupational stress among most nurses [42]. The heavy workload and high incidence of clinical emergencies result in prolonged mental strain. The constant consumption of psychological resources for self-regulation leads to a depletion of these resources [43], which in turn induces burnout and negative emotions such as anxiety and depression, significantly impacting nurses’ physical and mental well-being [44].

The higher the academic degree, the higher the workaholism levels, which contradicts previous findings [38]. They concluded that workaholism levels are higher among nurses with specialized degrees than those with bachelor’s degrees, hypothesizing that specialized nurses must continuously learn to enhance their skills to remain competitive [38]. Workaholism tendencies among highly educated nurses may vary depending on their occupational roles. For instance, nurse managers often bear leadership responsibilities requiring 24/7 emergency response capabilities, while also facing dual pressures from clinical supervision and administrative coordination duties [45]. Furthermore, the widespread adoption of mobile work platforms allows for constant work accessibility, further blurring work-life boundaries. These role-specific factors may serve as significant drivers of workaholism among highly educated nurses.

Almost all definitions of workaholism incorporate the idea that workaholics work longer hours than the average person [5, 46]. Workaholism is characterized by an addictive tendency to work long hours driven by an internal compulsion that is difficult to control, frequently thinking about work during non-work hours, and overworking beyond what is reasonably required, even when it leads to negative consequences. Currently, the shortage of nursing human resources persists, along with an imbalanced nurse-patient ratio. The more weekly working hours nurses have, the less time they can dedicate to family life, exacerbating family-work conflict and making the tendency toward workaholism increasingly pronounced [15]. Fundamentally, this phenomenon may stem from the influence of Confucianism and collectivism in traditional Chinese culture, which emphasizes collective goals over individual well-being. In such a context, society often celebrates those who overcommit to work as role models.

Higher job titles and positions were associated with a higher likelihood of workaholism. Benner’s theory suggests that nurses progress through five stages: novice, advanced beginner, competent, proficient, and expert [47]. Furthermore, professional competence gradually improves across these stages. Therefore, nurses with greater competence may assume greater responsibilities and pressures.

Nurses with children showed significantly higher workaholism scores than colleagues without children (*β* = 0.06, *P* = 0.013). We posit that this phenomenon may be partially explained by factors such as: (1) financial pressures from childcare costs, (2) emotional needs to provide stability for children, and (3) internalized societal expectations to model diligence. Furthermore, future research should explore the occupational characteristics within different nursing subgroups. Notably, our findings align with neurobiological evidence suggesting that parenting-specific stressors may increase susceptibility to addictive behaviors [48].

This study provides actionable insights for nursing administrators from both clinical and research perspectives. In practice, nursing managers should prioritize high-risk nurse populations through early identification of work addiction tendencies and implementation of proactive interventions. Future initiatives should adopt targeted measures across dual dimensions: At the individual level, cognitive restructuring should be utilized to enhance nurses’ self-capacity appraisal accuracy, complemented by job crafting strategies and leisure engagement to alleviate stress, ensure adequate recovery time, and facilitate transition toward a healthy, engaged work state. Organizationally, tailored career development plans should be formulated, workloads judiciously optimized, and structural support provided for work-leisure balance realignment to mitigate job demands’ detrimental effects on work-addicted nurses. Furthermore, rigorous validation of these interventions’ effectiveness and their impacts on patient safety outcomes should be conducted.

This study has several limitations. First, it did not comprehensively examine the impact of department-specific factors on workaholism. Second, the analysis was limited to the relationship between workaholism and demographic variables, whereas antecedent variables (such as personality traits, job demands, social support, and other organizational factors influencing nurses’ workaholism) warrant further investigation. Despite these limitations, our findings may still offer valuable insights for nursing administrators.

## Conclusion

The results of the study showed that Chinese nurses exhibited moderate levels of workaholism, with higher scores in excessive work tendencies. In addition, this study provided evidence that education, title, position, night shifts per week, weekly working hours, and parenthood status can significantly affect the level of workaholism among Chinese nurses. These findings offer practical insights for mitigating excessive workloads. Nursing managers should focus on these high-risk populations, monitor their dynamic workaholism levels, and actively guide nurses to reduce their workload through rational human resource allocation. This approach will help maintain their physical and mental well-being and promote the healthy development of the nursing team.

## Electronic supplementary material

Below is the link to the electronic supplementary material.

Supplementary Material 1

## Data Availability

The authors confirm that the data supporting the findings of this study are available within the article [and/or] its supplementary materials.
