# Supplementary material for: Workaholism among nurses in China: a nationwide cross-sectional survey
Source: BMC Nurs. 2025 Jul 1;24:676. doi: 10.1186/s12912-025-03455-5 (PMC12211388; doi:10.1186/s12912-025-03455-5)
Supplement: Supplementary file 1 — Supplementary Material 1 [file 12912_2025_3455_MOESM1_ESM.docx]

**Supplementary File 1.** Probability plots for continuous variables (Fig. 1).

Quantile-quantile plots (Q–Q plots) for the variables: DUWAS score


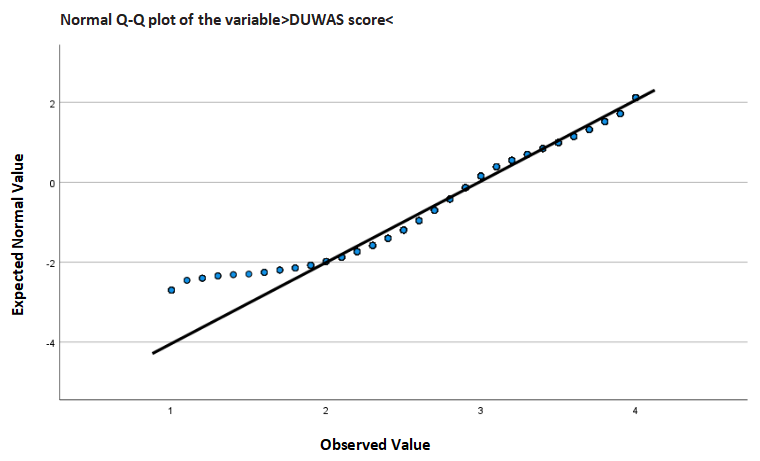


**Figure 1.** Q-Q Plot of the variable DUWAS score.
